# Supplementary material for: Visual–acoustic thigmotaxis in zebrafish larvae: a high throughput NAM for neurotoxicity assessment
Source: Front Toxicol. 2026 May 4;8:1753174. doi: 10.3389/ftox.2026.1753174 (PMC13180826; doi:10.3389/ftox.2026.1753174)
Supplement: Supplementary file 1 [file Supplementaryfile1.docx]

**Visual–Acoustic Thigmotaxis in Zebrafish Larvae: A High throughput NAM for Neurotoxicity Assessment**

SUPPLEMENTARY MATERIAL


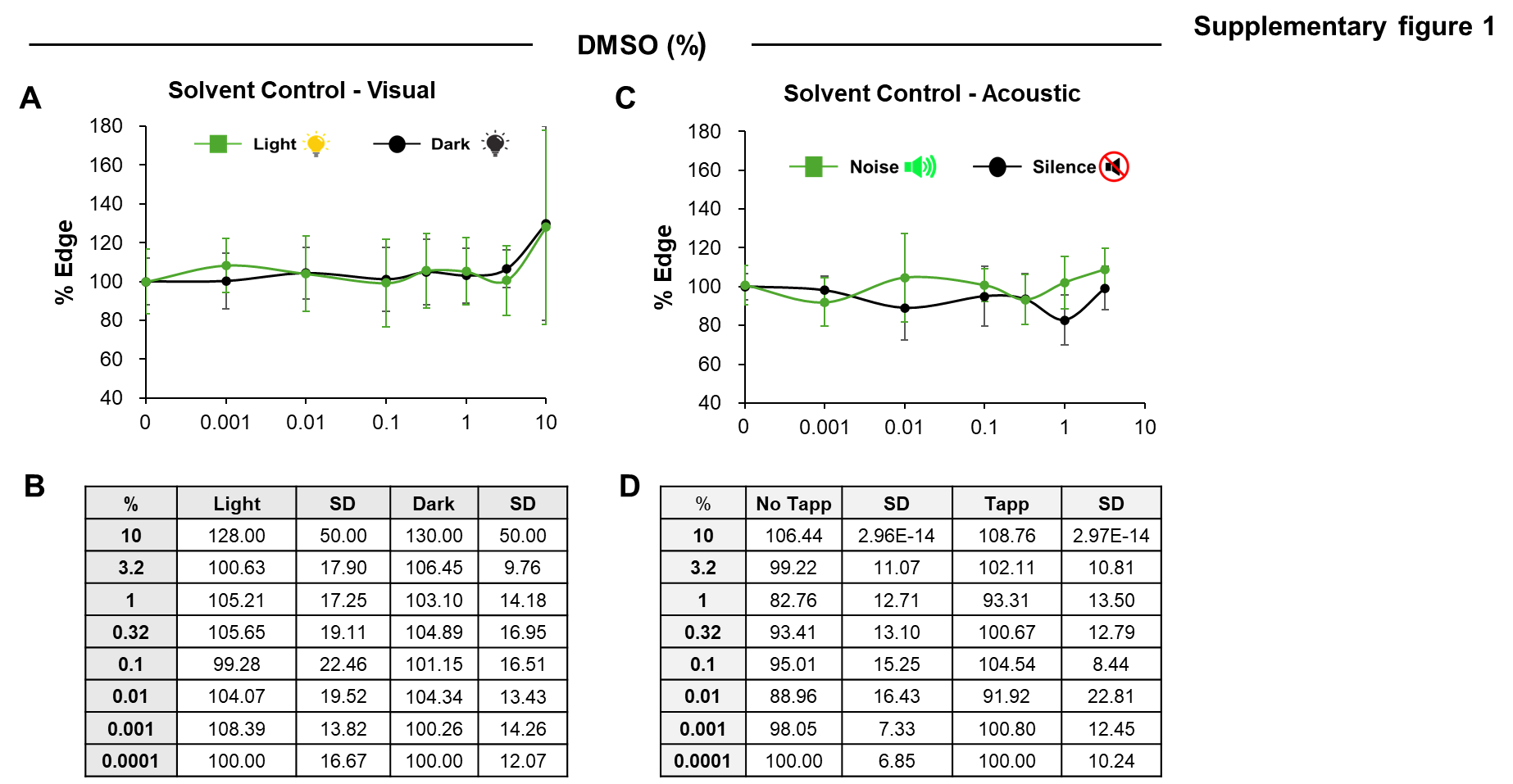


**Figure A.1:** Tests of solvent (DMSO) concentrations to evaluate maximum doses in thigmotaxis assay in both visual and acoustic (n=12) modes in 24/96 well plates. Panel (A) and (B) depict graphic representation and values of the percent distance in the edge by the larvae for the edge of the plates after visual stimuli (light in green, n=36; dark in black, n=36). Panel (C) and (D) depict graphic representation and values of the percent distance in the edge by the larvae for the edge of the plates after acoustic (noise in green, n=12; silence in black, n=12).


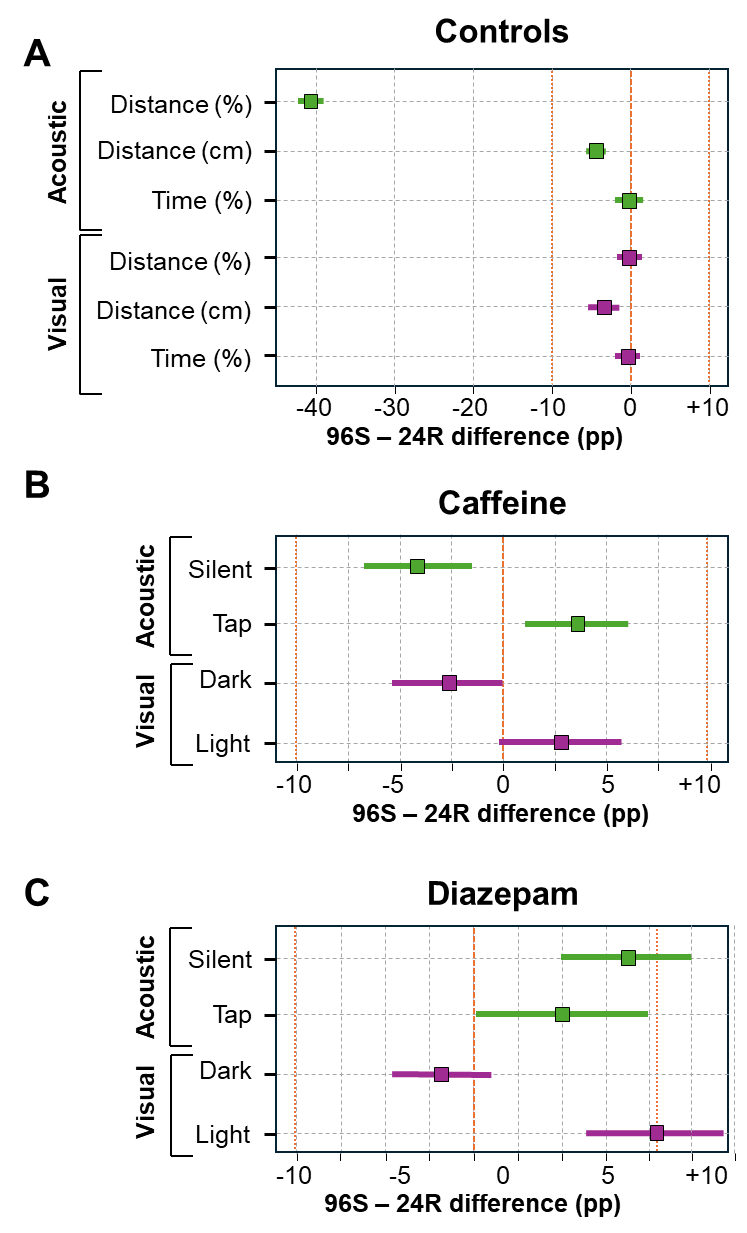


**Figure A.2. Representation of 96S–24R plate differences using Forest/TOST plots** (Δ = ±10 percentage points). Each point shows the pooled mean difference (96S − 24R) and the horizontal bar is the 90% CI. The vertical orange dashed line marks 0 (no difference) and the orange dotted lines mark the equivalence bounds at ±Δ (±10 percentage points).. A) Controls endpoints include Time (%), Distance (%), and Distance (cm) at Edge. Visual stimuli are Light/Dark and acoustic stimuli are Tap/Silent (pooled). Caffeine (B) and diazepam (C) endpoints are Time % (Edge). Values are pooled across concentrations within each assay/condition using fixed-effect inverse-variance meta-analysis with Welch/Satterthwaite uncertainty. A comparison is classified as Equivalent when the entire 90% CI lies within ±Δ; if the CI extends beyond these bounds, the result is not equivalent and is further labeled Different when the two-sided Welch test is significant (p < 0.05), otherwise Inconclusive. Abbreviations: TOST, two one-sided tests; pp, percentage points; CI, confidence interval; Welch, unequal-variance t-test.


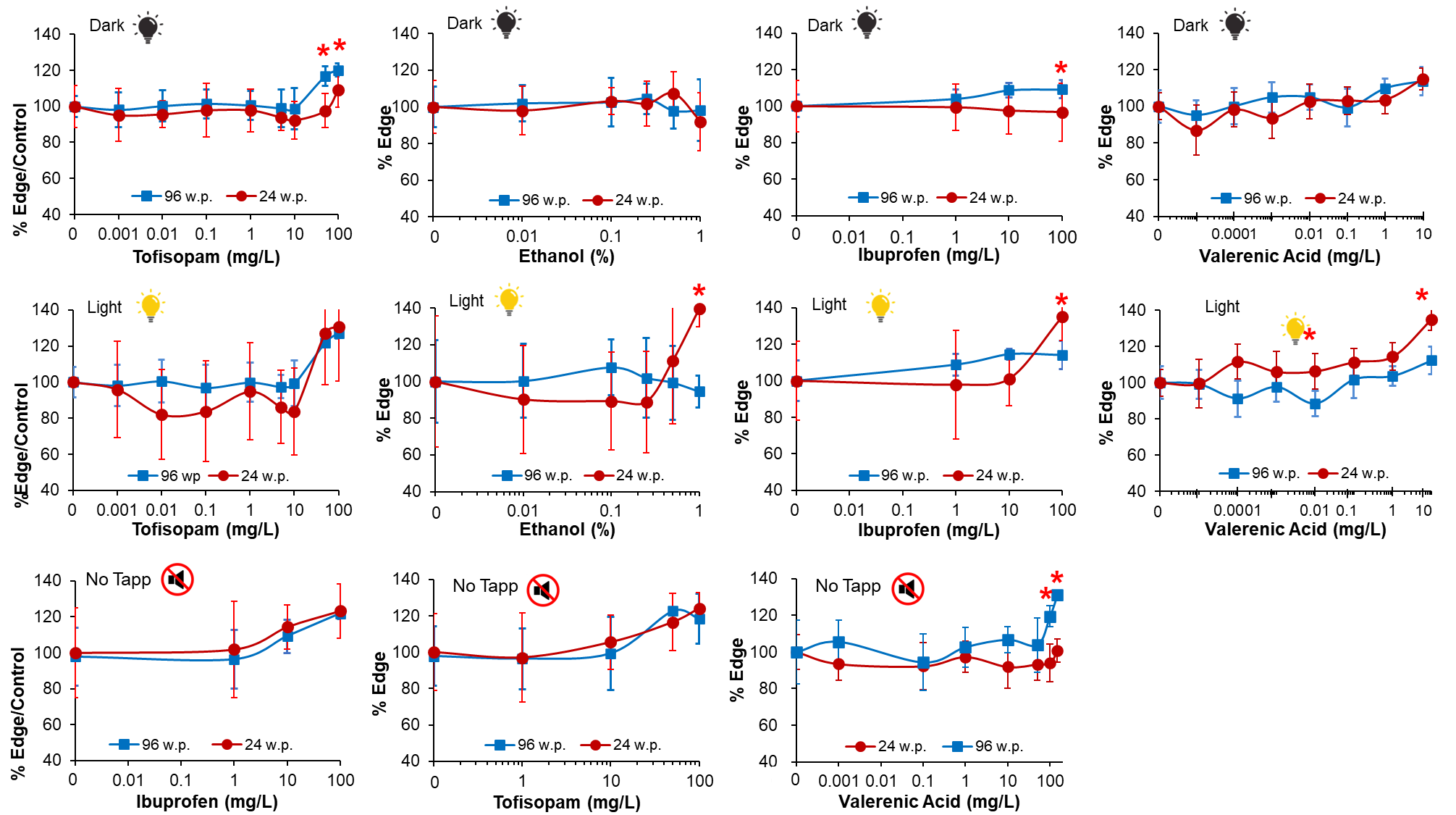


**Figure A.3:** Plate-format comparison for % time at edge (thigmotaxis) relative to control in 120 hpf larvae. Larvae were exposed for 1 h at 120 hpf and tested in 96-well square plates (96S; blue) and 24-well round plates (24R; red). Curves show the percentage of time spent at the edge normalized to the corresponding control for each concentration. For tofisopam, ibuprofen, and valerenic acid, responses are shown during dark (dark light-bulb icon), light (light-bulb icon), and silence following tapping (muted-speaker icon) periods. For ethanol, only dark and light periods are displayed. Symbols indicate mean ± SD. Asterisks above points denote significant differences between plate formats at the same concentration and stimulus (p ≤ 0.05).


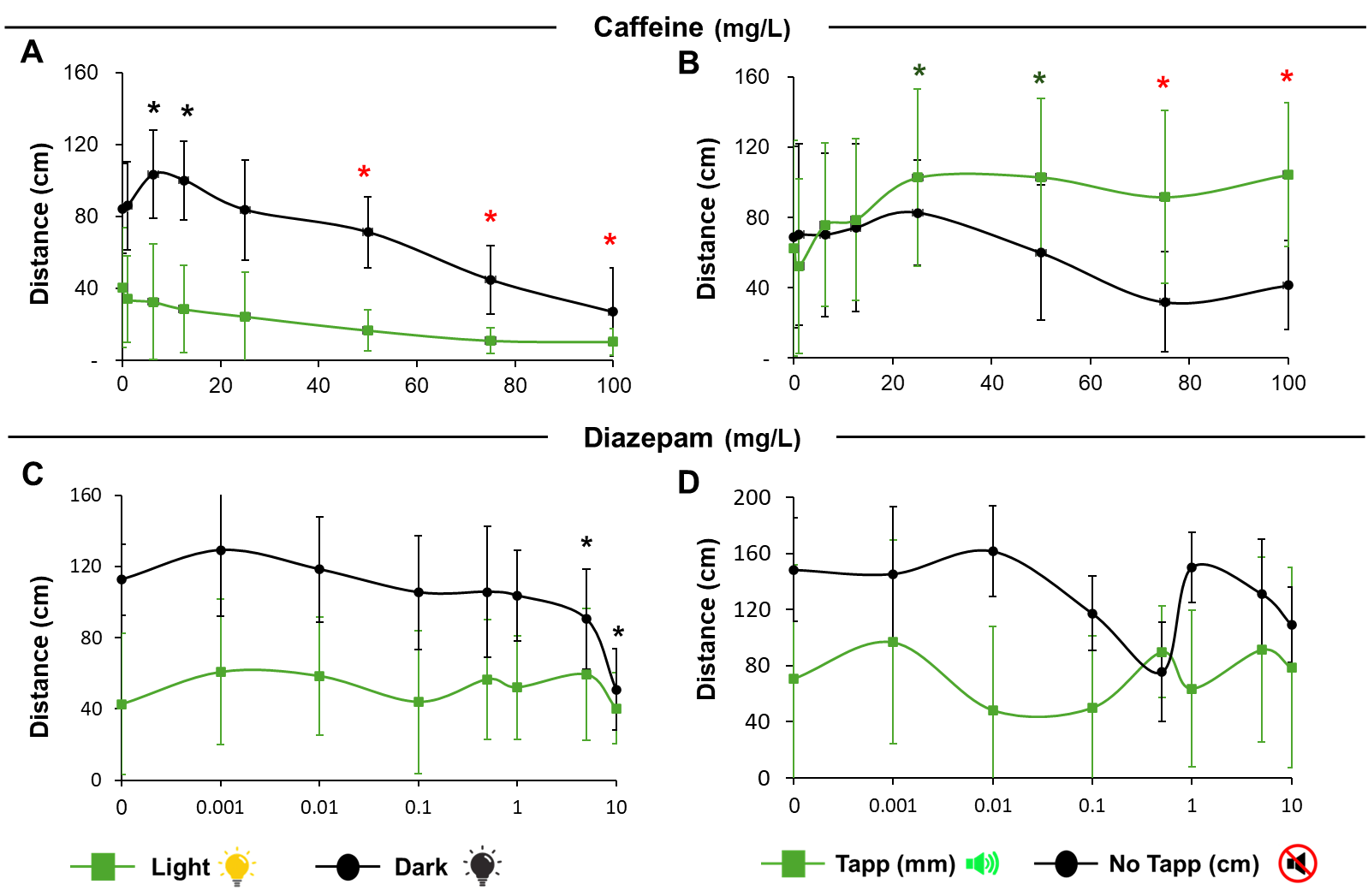


**Figure A.4**: **Results of total distance moved by larvae exposed to Caffeine (top panels) and Diazepam (bottom panels) in 96S well plates after visual (left) and acoustic (right) stimulation.** Green lines depict light or tapping periods and black lines depict dark or quite (No tapping) periods. Green asterisks above point denote significant differences of substance concentration vs control during light/tapping period. Black asterisk above points denotes significant difference of substance concentration vs control during dark/quiet period. Red asterisks above points denote significant differences of substance concentration vs. control for both periods. Data are presented as Means +/- SD.
